# A concept analysis of children with complex health conditions: implications for research and practice

**DOI:** 10.1186/s12887-020-02161-2

**Published:** 2020-05-26

**Authors:** Rima Azar, Shelley Doucet, Amanda Rose Horsman, Patricia Charlton, Alison Luke, Daniel A. Nagel, Nicky Hyndman, William J. Montelpare

**Affiliations:** 1 Psychobiology of Stress and Health Lab, Psychology Department, Mount Allison University, 49A York Street, Sackville, NB E4L 1C7 Canada; 2 NaviCare/SoinsNavi, Sackville, Canada; 3 Department of Nursing and Health Sciences, University of New Brunswick, PO Box 5050, Saint John, NB E2L 4L5 Canada; 4 Interdisciplinary Studies, School of Graduate Studies, University of New Brunswick, 100 Tucker Park Rd, Box 5050, Saint John, NB E2L 4L5 Canada; 5 Faculty of Nursing, University of Prince Edward Island, 550 University Avenue, Charlottetown, Prince Edward Island C1A 4P3 Canada; 6 Veterans Affairs Canada, PO Box 7700, Charlottetown, PE C1A 8M9 Canada; 7 Faculty of Science, University of Prince Edward Island, 550 University Avenue, Charlottetown, Prince Edward Island C1A 4P3 Canada

**Keywords:** Concept analysis, Definition, Children, Complex, Health conditions, Chronic

## Abstract

**Background:**

This concept analysis aimed to clarify the meaning of “children with complex health conditions” and endorse a definition to inform future research, policy, and practice.

**Methods:**

Using Walker and Avant’s (2011)‘s approach, we refined the search strategy with input from our team, including family representatives.

We reviewed the published and grey literature. We also interviewed 84 health, social, and educational stakeholders involved in the care of children with complex health conditions about their use/understanding of the concept.

**Results:**

We provided model, borderline, related, and contrary cases for clarification purposes. We identified defining attributes that nuance the concept: (1) conditions and needs’ breadth; (2) uniqueness of each child/condition; (3) varying extent of severity over time; 4) developmental age; and (5) uniqueness of each family/context. Antecedents were chronic physical, mental, developmental, and/or behavioural condition(s). There were individual, family, and system consequences, including fragmented services.

**Conclusions:**

Building on previous definitions, we proposed an iteration that acknowledges the conditions’ changing trajectories as involving one or more chronic condition(s), regardless of type(s), whose trajectories can change over time, requiring services across sectors/settings, oftentimes resulting in a lower quality of life. A strength of this paper is the integration of the stakeholders’/family’s voices into the development of the definition.

## Background

Although parents, clinicians, and researchers recognize a complex health condition in a child, establishing a unified definition of “children with complex health conditions” (CCHC) is challenging [1]. However, reaching a clear, full consensus definition about the concept of CCHC is necessary to inform research, policy, and practice [1]. A concept analysis is a critical step to advance our understanding of a given concept. A common understanding of the concept of CCHC is vital to improving these children’s quality of care and life.

To our knowledge, our paper is the second concept analysis of CCHC. Indeed, the first systematic concept analysis of “children with complex care needs” [2] was conducted based on Rodger’s evolutionary method. As data sources, Brenner et al. [2] used PubMed, PsycINFO, and the Cumulative Index to Nursing and Allied Health Literature. They reviewed 140 articles and found that CCHC refer to multi-faceted health and social care needs, often with a medical diagnosis or, at times, without a formal diagnosis. They concluded that the concept of “children with complex care needs” implies that children’s needs follow a chronic and dynamic nature, across settings whilst being influenced by family and healthcare systems. In contrast to Brenner et al. [2], our concept analysis is focused on “children with complex health conditions” and takes a comprehensive approach using Walker and Avant (2011)‘s method [3]. The latter is a different approach, which includes broader data sources, namely both the literature on CCHC and information from interviews with key stakeholders on the meaning of this concept.

Regardless of the specific approach used, it is already known that a widely used definition of CCHC [4] was developed by the 1998 Maternal and Child Health Bureau (part of the Health Resources and Services Administration, which is a division of the U.S. Department of Health and Human Services). According to this definition, “children and youth with special health care needs (CSHCN)” involve pediatric patients who either have or are at increased risk for chronic physical, developmental, emotional or behavioural conditions and who also need physical health care and other services (e.g., education, mental health and/or social services) of either a type or an extent beyond that required by the general population of children [4]. Using this definition, it was found that one in five U.S. families has a child or youth with a special health care need [4]. Some of these children may have severe care needs whilst others may show special health care needs without receiving any medical diagnosis. The age range of CCHC varies, generally including children from 0 to 18 or 19 years of age.

Keeping this in mind, defining CCHC is further challenging because there are many related terms, including “technology-dependent children”, “children with special needs” or “children with multi-system chronic disease processes” [4, 5]. These categories are complicated by the: (1) possible comorbidity of conditions [6]; and (2) various needs stemming from different conditions. To complicate matters further, related terms are often used interchangeably, and sometimes CCHC are placed in distinct categories of related terms.

In a systematic review (2007), van der Lee et al. [7] performed a PubMed and a Web of Science search, in addition to a hand search, and found 64 English and Dutch articles on pediatric chronic health conditions. These articles needed to report a conceptual definition and/or an operationalization of children’s (aged 0–18) chronic health conditions. Van der Lee et al. found a range of different definitions of “chronic health conditions”, yielding a wide variability in their prevalence rates (0.22–44%). Only four of these identified definitions of “chronic health conditions” were cited commonly in the articles. Of note, the authors concluded that there was a need for an international consensus on a definition [7]. Brenner et al.’s (2018) concept analysis was a significant step toward the full establishment of such a definition [2]. Building on these two key papers, our concept analysis applied the rigorous process of Walker and Avant’s method [3] to further clarify the concept of CCHC. This paper contributed to the literature by (1) confirming existing definitions and (2) building on/extending the latter with a new proposed iteration.

## Methods

The prevalence estimates of CCHC varied from 0.22 to 44% [7]. This wide range of estimates depended on different operationalizations of definitions in addition to multiple informants, types of data, and years of publication. Providing a conceptual framework for research and clinical purposes, Walker and Avant’s (2011) method [3] guided our concept analysis on CCHC. This approach is the most widely used in the literature, and involves a comprehensive interdisciplinary review, especially useful with complex and intersectoral concepts like CCHC. Walker and Avant’s method [3] combines the analysis of the literature along with other data sources, including qualitative data from interviews/focus groups [8]. We followed Walker and Avant’s (2011) eight steps [3]:

1. Selecting a concept: To inform research, policy, and practice, we selected the concept “children with complex health conditions” (CCHC). We focused on this concept because it is broad, including all types of physical, mental, neurological, and/or behavioural conditions or disabilities, with or without diagnosis. CCHC’s needs may or may not emerge from chronic illnesses, symptoms’ severity, clinical care, or from possible comorbidity of conditions. Care needs are considered complex when compared to the needs of the general population of children.
2. Determining the purpose of the analysis: We determined the purpose of the analysis, which was to clarify the meaning of CCHC.
3. Identifying uses of the concept: This step identified the use of the concept and related terms, integrating findings from the published and grey literature, and from stakeholder interviews.
4. Determining the defining attributes: Similar to signs or symptoms, defining attributes are critical characteristics that assist in clarifying concepts and differentiating them from their related concepts [3]. This fourth step described the characteristics most commonly related to CCHC in the family representatives’ perspectives and in the literature.
5. Identifying the model case: This step includes all the defining attributes of CCHC from step 4.
6. Identifying borderline, related or contrary cases: Identifying these cases aims to further refine the concept of CCHC. Borderline cases contain some defining attributes of the CCHC concept identified in step 4, but not all of them. Related cases are somehow related to the CCHC concept. They may have some of its defining attributes but perhaps not the ones considered more critical. Contrary cases are not the concept.
7. Identifying antecedents and consequences: Antecedents are the concept precursors whereas consequences are the concept outcomes.
8. Defining empirical referents: This last step consists of identifying the empirical referents of the defining attributes of a concept, which describes its practical expression.

### Literature search

First, to refine our literature search strategy, we consulted the following family representatives through individual interviews: (1) a young adult with behavioural and mental health care needs; (2) a father of a child who was born prematurely and has a severe mental health condition; (3) a mother of two CCHCs, one with a rare genetic disorder; (4) a librarian, who is also a mother, who grew up as a child with complex needs; and (5) the rest of our team, including researchers and clinicians. Informed by their input, we conducted a published and grey literature search.

Second, we conducted a search of key terms in PubMed, PsycINFO, CINAHL, Science Direct, and ERIC databases, as well as a Google Search of the grey literature. We initiated our search process in January 2016, completed it in January 2017, and updated it in August 2018. The key terms included: (“complex health conditions” OR “complex chronic conditions” OR “medical complexity” OR “complex health needs”) in conjunction with (“infant” OR “neonate” OR “newborn” OR “toddler” OR “children” OR “child” OR “teen” OR “youth” OR “adolescent” OR “pediatric”) AND (“define” or “definition” or “defined as”). We included abstracts of articles published in English or French that focused on CCHC, 0–19 years. We only included those explicitly reporting on the definition of CCHC.

Third, we restricted the final analysis to articles published after van der Lee et al. (2007)‘s landmark systematic review from December 2006 [7].

### Grey literature search

We performed a google search with the following key terms: intitle: (“children with complex health conditions” OR “children with complex chronic conditions” OR “children with medical complexity”) AND “definition”. We searched the following government sites: (gc.ca OR gnb.ca OR on.ca OR ab.ca OR bc.ca OR mb.ca OR nl.ca OR ns.ca OR pe.ca OR qc.ca OR sk.ca OR yk.ca OR nu.ca OR nt.ca). We considered .org websites and specific organizations, as needed.

### Stakeholder interviews

The study participants consisted of a convenience sample of 84 stakeholders. In line with Walker and Avant’s (2011) method [3], we conducted phone interviews with key stakeholders involved in the care of CCHC in two Canadian provinces: New Brunswick (NB) and Prince Edward Island (PEI). Developed by our research team, phone interviews were conducted by a research assistant using open-ended questions that lasted for about an average of 20 min. Examples of the semi-structured interview questions were: (1) “Who would you identify as children with complex health conditions”?; (2) “In your opinion, what are the distinct needs of these children and their families?”; and (3) “From your perspective, what are the specific or unique services that these children require?” We analyzed data using Braun and Clarke’s (2006) six phases of qualitative thematic analysis [9], which we fully described elsewhere [10]: (1) familiarize self with data, (2) generate the initial codes, (3) search for themes, (4) review the themes, (5) define and name the themes, and (6) provide a report. Under the guidance of three of this paper’s co-authors (RA, SD, WM), four members of the team, coded the transcripts from the first three phone interviews to generate preliminary codes and working definitions; this guided subsequent analysis of the remaining phone interview transcripts. Data management was done utilizing NVivo 10™software. This study was conducted according to Helsinki Declaration and approved by the Research Ethics Boards of all the co-authors.

## Results

### Steps 1–3: selecting a concept and determining the purpose of the analysis

These first two steps were described in the methods section.

#### Published literature

Our search resulted in a total of 1300 articles. After removing duplicates and articles in other languages, we evaluated 772 articles for inclusion. We performed two levels of screening. In the first screening, we reviewed bibliographic information with the titles and abstracts. In the second screening, we reviewed the full-text articles for those articles that met the inclusion criteria. The final number of included articles in the analysis was 77.

The types of articles included in this concept analysis were empirical studies, reviews, and clinical cases. Specifically, there were 26 quantitative studies, 17 qualitative studies, 7 studies with a mixed-methods design, 7 randomized control trials, 2 cases studies, 12 review of the literature, 2 meta-analyses, 2 meta-synthesis, and 2 systematic reviews. The samples used ranged from children of all ages to parents, including mothers or fathers. The children’s care needs were also diverse in terms of type and complexity (e.g., chronic pain, cystic fibrosis, mental health conditions, type I diabetes mellitus, congenital heart disease, rheumatoid arthritis, Duchenne muscular dystrophy, cancer, ventilator-assisted, etc.). Studies took place in different countries, including the USA, Canada, Iceland, France, Sweden, the Netherlands, South Korea, Germany, Taiwan, New Zealand, Israel, Palestine (i.e., Gaza), etc.

We focused on the many terms for CCHC, which are often used interchangeably, leading to confusion. The identified terms were extracted from the 77 included studies, with the two most common being (about 40 times): “children with special health care needs” and “children with chronic health conditions”.

Overall, the terms found included children with special health care needs, complex chronic conditions, and medically fragile children, among others.

First, and since 1998, the Maternal and Child Health Bureau (MCHB) has defined “children with special health care needs” (CSHCN) as having or being at increased risk of having a chronic physical, mental, developmental, or behavioural condition, requiring health and related services of a type or amount beyond that required by children generally [11].

Second, another commonly used term for CCHC was “complex chronic conditions” (CCCs) [12–17], defined as involving one or more chronic conditions [18]. In studies [19–22] that drew on Feudtner et al. [12, 23], CCCs were conditions that may be expected to last at least 12 months, unless death occurs before, and to involve different organ systems or one organ system severely enough to require speciality care or hospitalizations. In a theoretical paper [13] and in empirical studies [14, 17], the authors focused on children with “life-threatening” [17] and “life-limiting” conditions [14], forming a sub-sample of CCHC, namely those with medical complexities and in palliative care. A doctoral dissertation on palliative care used terminal and life-threatening illnesses interchangeably [15]. Similarly, in a book on palliative and end of life care, Grinyer and Barbrachild [16] resorted to the terms “self-limiting” and “life threatening illnesses” as a sub-category of CCHC. Although this term is usually applied to cancer diagnoses, the focus may be HIV/AIDS [21]. Other definitions focused on palliative conditions that could be either life-threatening (cure is possible) or life-limiting (no hope for a cure) [22].

Third, there was a sub-group of “medically complex” or “medically fragile” children [19], including those with intense needs due to multisystem conditions, technology dependence, or complex treatments. These CCHC, the most medically complex cases [24]. Hall [25] p. 179, were considered to have “medically complex chronic disease” or even “multisystem chronic disease”. Both terms refer to the “involvement with multiple subspecialists and medical technology or device dependence” (e.g., gastrostomy tubes, tracheostomies, etc.). Some studies focused on specific conditions, such as cerebral palsy [26], hemophilia A, B or Factor VII (FVII) deficiency [27], or medical complexities with *a common denominator,* such as incontinence [28].

In sum, there were many terms for CCHC and related terms that were used interchangeably, which may cause confusion (see Table 1). Implicit in the definition of “children with special health care needs” by the 1998 Maternal and Child Health Bureau [11] is the uniqueness of each child, family, and condition, whether diagnosed or at risk of a condition. The term “complex chronic conditions” recognized the varying extent of severity due to comorbidity. Finally, in the term “special health care needs children” emphasis was put on the child’s developmental age, which refers to their age of functioning physically, socio-emotionally, or cognitively.

**Table 1:** A list of the identified terms extracted from the 77 included studies

Extracted terms	Categories of the terms	Used terms in the literature	Relevant articles
Terms/related terms for children with complex care needs (CCHC)	Complex care needs	Children with special health care needs^a^	Across the studies
		Children with chronic health conditions^a^	Across the studies
		Children with complex care needs	2
		Children with special needs	4, 5, 24, 33
		Children with chronic physical illness	6
		Children with complex health conditions	7, 10
		Children with special health care needs (CSHCN)	11, 31
		Complex chronic conditions (CCCs)	12–17, 19–22, 23, 43
		Children with life-limiting conditions	13, 14 15
		Children with life-threatening conditions	13, 15, 17
		Children with life-threatening illnesses	16, 22
		Children with self-limiting conditions	16, 22
		Children with palliative conditions	22
		Children with special health care needs	36
		Children with chronic conditions	39
		Children with complex chronic conditions	40
		Children with chronic illnesses	42
		Children with severe neurodisabilities	45
	Specific conditions	Children with cancer	13, 48
		Children with cerebral palsy	26
		Children with hemophilia A, B or Factor VII (FVII) deficiency	27
		Children with non-traumatic dental conditions	41
		Children with autism	47
	The most complex cases (sub-category of CCHC)	Technology-dependent children	4, 5
		Children with multisystem chronic disease processes	4, 5, 25
		Children with medical complexity	4, 11, 19, 37, 46
		Medically complex children	5
		Children medically fragile	19, 24
		Medically fragile children	24
		Children with medically complex chronic disease	25
		Children with medical complexities with a common denominator (e.g., incontinence)	28

^a^ these terms were the two most common (about 40 times)

#### Grey literature

The Google search resulted in 546 hits, and all were reviewed. The grey literature found consisted of North American governmental websites and/or non-governmental organizations (i.e., Canadian provinces of Ontario and Prince Edward Island as well as the United States of America) and identified 7 definitions.

Overall, some of the North American governmental websites and/or non-governmental organizations (Canadian provinces, American states) considered CCHC to include children who have chronic conditions when they last over 6–12 months [29, 30]. In Canada, using the MCBH’s definition [11, 31] that was endorsed by van der Lee et al. [7], the PEI’s Minister of Health and Wellness developed a definition of *children with special needs* as follows: “Children and youth up to 18 years of age and their families who require significant additional health, social, environmental, educational support–beyond that which is required by children in general–to enhance or improve their health, development, learning, quality of life, participation, and community inclusion” ([32] p. 2]).

In contrast to this broad definition [33, 11], the Complex Care for Kids Ontario strategy ([34] p. 1) used a standard operational definition for the sub-group of medical complexity, referring to these CCHC as “technology dependent and/or users of high intensity care, fragility, chronicity and complexity”. These conditions last at least 6 months but death may occur earlier [33]. The Children’s Hospital Association [29], which represents over 220 American children’s hospitals, used the terms “medically complex children” or “children with medical complexities” or “children with medically complex conditions” to refer to highly demanding pediatric patients [30]. Based on the 3M™ Clinical Risk Groups [34], which is a classification system and risk-adjustment tool that measures burden of illness, CCHC may fall into five categories, including non-chronic, episodic chronic (12 months long and likely episodic; not likely lasting into adulthood), lifelong chronic, and complex chronic or malignancies. There may be chronic conditions in one/more body systems or a single dominant condition [35].

In sum, CCHC’s conditions were persistent in either one dominant illness/disability or more bodily systems, lasting beyond 6 or 12 months. CCHC was used as a broad concept, including cases with dependency on technology, high intensity care use, and with highly complex or chronic conditions (fitting with the published literature’s medical complexity).

#### Stakeholders

There were 42 health, 22 social, and 20 educational stakeholders. Health stakeholders included pediatricians, occupational therapists, physiatrists, physiotherapists, nurses, and speech language pathologists. Social stakeholders included social workers and allied professionals. Educational stakeholders included teachers and special educators.

In descending order, the most commonly reported elements (aspects or parts of) of the definition of CCHC as used by the stakeholders were as follows:

1. A single definition for all types of conditions, including physical/organic (e.g., brain diseases), mental/addictions, and behavioural conditions that require management that is “long-term” or “on-going”, across the “lifespan” or “lifelong” (*n* = 84);
2. Needs that require support from a high number of professionals, including those across sectors such as social services (*n* = 62);
3. Severity (*n* = 52) in the form of the complexity of conditions (involving one or more bodily systems) or impaired quality of life, resulting in family stress or additional developmental needs. Some stakeholders (*n* = 26 of 52) added: all the defining elements may apply at one time but not at another (e.g., remission); Others (*n* = 16 of 52) added that it is probably more difficult to identify cases involving less severe cases and that a definition would need to capture these cases.
4. Strictly medical organic/physical problems (*n* = 17), in contrast with the most commonly reported element around the need for a broader lens (see 1 above); and
5. A broad range of age at the time of diagnosis, starting from conception (*n* = 14);

Less commonly reported elements of the stakeholders’ definitions of CCHC were as follows:

1. Functional problems where CCHC may feel better at times (*n* = 3);
2. A forgotten positive connotation of the term “chronic conditions” where children now generally live longer with long-term management (*n* = 2);
3. Limited school attendance (n = 2);
4. Difficulty of receiving support, regardless of the conditions’ degree of severity (n = 2);
5. The need to travel for care (*n* = 1),
6. The need to not have a vague broad definition (n = 1);
7. The inclusion of chronic pain (n = 1)
8. A focus on medical frailty (n = 1);
9. Families’ own appraisal of conditions and coping (n = 1); and
10. Refugee-related needs (n = 1) where CCHC’s conditions may become more complex because of the need for an interpreter in schools, a lack of knowledge of medical background, and complex needs (n = 1).

Many stakeholders reported a need for a broader definition than the one developed by McPherson et al. (or MCHB) [11, 31] that includes:

(1.1) chronic conditions that endure for at least 6 months; or
(1.2) cognitive learning disabilities, especially without early support and where the child can develop mental illnesses;
(1.3) conditions that do not respond to treatment;
(1.4) short-lived conditions (when severe enough);

### Step 4: determining the defining attributes

First, the family representatives we consulted raised the following points that were included in our search strategy: (1) the concept of “CCHC” would not be complete without the inclusion of “psychosomatic conditions” (involving the mind and body); (2) a broad definition of CCHC because of commonly comorbid conditions; and (3) the term “children with complex health needs” would capture CCHC’s unique and complex daily life.

Second, to frame the defining attributes of CCHC, we drew on the existing definitions of CCHC and of related concepts, which were referred to in Step 3 and summarized herein as follows: *children with special health care needs* [CSHCN] [26, 36–39], *children with special school health needs*, *children with complex chronic conditions* [CCCs] [12, 23, 40–42] or *children with pediatric chronic conditions* [7, 43], *life-threatening* and *life-limiting conditions* [5, 44], *children with medical complexity* (CMC) (e.g., children with severe neuro-disabilities [4, 45–47], and *particular conditions*, including genetic or developmental ones [48].

Third, in their systematic review [7], van der Lee et al. (2007) defined “complex chronic conditions” as “commonly requiring multidisciplinary input”. In some studies in van der Lee et al.’s systematic review [7], CCHC were defined by their developmental stage. Bearing this in mind, one must consider that human development is embedded in a broader socio-cultural context, as described in those studies. For CCHC, this context includes factors such as their immediate/larger families, schools, neighborhoods, communities, culture, and peers, among others. For example, a child develops as whole but also simultaneously in domains (physical, motor, socio-emotional, cognitive, etc.). This development is fostered by a loving family, through relationships with parents, siblings, relatives, neighbours, and community members, among others. In addition, a child has relationships with siblings, peers, school educators, and other adults.

Finally, to determine the defining attributes of the concept of CCHC, we integrated the findings from stakeholder interviews and the literature [2, 7]. To do so, we followed three steps: (1) Documenting all repeatedly occurring attributes from the literature; (2) Reducing our exhaustive list of attributes to a concise yet complete list by grouping common points together; (3) Comparing and contrasting this list with the interview data from the stakeholders. More frequently reported attributes became the defining attributes of CCHC, which were: (1) breadth of conditions, with or without diagnosis, possibly comorbid, resulting in multiple care needs and requiring services often across settings; (2) uniqueness of each child; (3) varying extent of severity at different time points; (4) importance of developmental age; and (5) uniqueness of each family’s context, namely within a broader socio-cultural context. Taken together, there were breadth and uniqueness of conditions, severity, and of required services, resulting in a need for broader definitions of CCHC.

### Step 5: identifying the model case

A typical example of CCHC is Mathew, a 7-year-old boy from a remote village in Canada who was born with a congenital condition that limits his mobility, delays his development, and impacts his daily life. His single mother must regularly leave her job for a few days to travel to a larger city for appointments with a physiotherapist, speech pathologist, and pediatrician. Occasionally, she must drive out of province to a children’s hospital for yet another surgery for her son. She worries about time off from her job. She is afraid of being fired as without her job it will be challenging to put bread on the table. Furthermore, she is concerned about the icy roads in the winter. She often feels lost, overwhelmed, frustrated or frightened whilst dealing with all the professionals taking care of her son. Confronted with fragmented services, she regularly needs to repeat Mathew’s story every time she meets with a new health care provider. She attends her son’s school on a regular basis to educate teachers about his condition and to advocate for his basic needs, such as easier physical access to a washroom. She often feels guilty about her son’s behavioural problems in school.

This model case includes all the defining attributes of CCHC determined in Step 4, which are as follows: (1) one or more chronic condition, requiring different services, namely physiotherapy, speech pathology, pediatrics, surgery, and school accommodation; (2) Uniqueness of the child and his condition (7-year-old boy, growing up with a condition in a remote village that requires multiple, long-distance services; (3) Varying extent of severity (birth condition that impacts daily life); (4) The importance of developmental age (limited mobility and delayed development); and (5) Uniqueness of family’s context (time off work/school, parental advocacy, effort to re-tell the child’s story due to fragmented services, and travels).

### Step 6: identifying any borderline, related, or contrary cases

#### Borderline case

Gabriel, a 2-month-old infant, was born moderately preterm at 34 weeks of gestation. His birth weight was 2000 g. After 4 weeks, he was fully ready to be discharged from the neonatal intensive care unit to finally go home. This became clear to his care team when he showed that he was able to breathe without any support. Gabriel was able to maintain a stable body temperature. He did not have signs of infection. He kept gaining weight steadily. He was bottle-fed. His uncles and aunts participated in his feeding sessions. Everyone lived within a 5-km distance from the large teaching children’s hospital in question. They were able to visit easily. At the time of the discharge to home, contrary to most parents of preterm infants, they did not feel apprehensive. Their son was physiologically stable. Gabriel had a smooth transition to a safe home where his loving parents were ready to take care of him. Thus, this was not a particularly complex case.

This borderline case possesses most, but not all, of the attributes of CCHC, as determined in Step 4. It is missing the breadth of conditions, namely the resulting multiple care needs, as there were no long-term implications, and the importance of developmental age. Although he was born prematurely, Gabriel is considered “mildly premature” on the spectrum of prematurity, from mild (33–36 completed weeks of gestation) to moderate (28–32 completed weeks of gestation) to extreme (< 28 completed weeks of gestation). He did not have any complication besides prematurity. By two months postpartum, Gabriel had enough time to “catch up” on any growth delay.

#### Related case

Paula, an 11-year-old girl, has allergies that can have moderate complications, such as sinusitis and eczema. Although potentially complex, these complications are not severe enough to cause an anaphylactic shock. Paula’s condition can be easily managed by avoiding the non-common allergy trigger, although this may not be always possible. There is no comorbidity. Despite the potential severity, Paula’s life is not in danger. However, her family gets worried as they live on a farm in a small, remote, rural community, lacking access to a family physician or nurse practitioner. This requires long distance travels for a consultation, which may be challenging in the winter.

This related case shares some of the defining attributes identified in Step 4, namely the varying extent of severity, although not to the point of an anaphylactic shock, and the need for services across settings, although only because of a lack of services and a complex geography. This case is missing the breadth of care needs and severity.

#### Contrary case

Maya, a 5-year-old child, from a small village suffered from norovirus-like symptoms. Norovirus is the most common form of foodborne illness in the world. Maya had a fever higher than 38.9 C (102 F). She became more irritable than usual. She had diarrhea and showed signs of dehydration. The virus has spread to others in her community. Thus, it was not very difficult for the family physician to diagnose it. The case was clearly not complicated or long-term. No specific medication was needed, as antibiotics are not effective against viruses. The physician explained to Maya’s parents that antibiotics’ overuse is problematic, contributing to antibiotic-resistant strains of bacteria. After 24 h, Maya was back to her normal self.

This contrary case does not possess any of the defining attributes of CCHC. Clearly, Maya’s health condition was acute, neither complex nor comorbid, and did not require medication. Her parents were not distressed, especially where her diagnosis was confirmed fast and they quickly saw signs of improvement.

### Step 7: identifying any antecedents and consequences

#### Antecedents

Based on the integrated findings from the literature search, the grey literature, and interviews with stakeholders, CCHC have one/more chronic physical, mental, developmental, and behavioural conditions. Antecedents refer to this broad range of conditions and, by extension, to risk factors. An example may be the combination of genes and environmental factors in juvenile arthritis. C*onsequences:* CCHC require multiple services, including health, psychosocial, educational, and counseling. Care is provided by many clinicians, including pediatricians, other physicians, nurses, physiotherapists, psychologists, and social workers, among others. There may be system consequences (i.e. fragmented services), affecting the quality of life not only of CCHC but also of their family and care team. For example, siblings may suffer from these consequences. They may feel tired from family travels or they may feel neglected if the parents’ attention is too focused on their CCHC.

### Step 8: defining empirical referents

The empirical referents, which are ways in which the CCHC concept can be practically observed or measured, were as follows: In general, as derived from both the literature and data from stakeholders, CCHC had chronic conditions that required complex care, showed high morbidity and possibly mortality, whilst experiencing increased healthcare system use [17]. Stated differently, CCHC were viewed as having challenges due to their more complex developmental and/or care needs. These challenges may be stemming from either chronic or severe/intense conditions, which may be diagnosed or not. As a result, they put more pressure on the healthcare system; some of them may even require complex clinical care. It may be straightforward to recognize a child with complex-to-very-complex conditions, namely in the context of medical complexity [11, 31]. However, identifying CCHC with less complexity becomes complicated because: (1) stakeholders define CCHC differently; (2) it is often harder to capture individual characteristics with population data sources than in one-on-one encounters; and (3) contrary to adults, CCHC often have a spectrum of heterogeneous conditions. Perhaps like adults, some CCHC may have a condition that becomes complex only when comorbid.

## Discussion

First, based on the integrated literature and clinical inputs from the stakeholders, the defining attributes of CCHC included the: (1) breadth of conditions and their resulting needs; (2) uniqueness of each child and condition, with or without a diagnosis; (3) varying extent of severity at different time points; (4) importance of context [11]; and (5) uniqueness of each family/family’s context [2].

Second, there were multiple terms related to the concept of CCHC, with the two most widely used terms including: (1) the MCHB’s *children with special health care needs* (CSHCN) [20] and (2) Feudtner et al.’s *complex chronic conditions* (CCCs) [18]. Other less commonly used related concepts were *life-threatening* or *life-limiting conditions* [13, 14, 17]. This sub-sample of CCHC [19, 20, 22, 24, 25] included medical complexities [14, 17] where children may die prematurely [21].

Third, we found multiple, and at times inconsistent, definitions in: (a) the published literature; (b) the grey literature; and (c) among the participating stakeholders. We showed the range of interpretations of the concept of CCHC and its related terms: CCHC’s conditions were chronic, even when they varied in their persistence, usually over 6 months to lifelong, unless death occurs prematurely. Some of CCHC-related definitions highlighted the severity of conditions. Despite its complexity, the concept of CCHC was nuanced by the: (1) breadth of conditions; (2) uniqueness of needs; (3) varying extent of severity at different points in times; (4) the child’s developmental age, and (5) uniqueness of families.

Fourth and finally, stakeholders expressed concerns with the two most widely used CCHC-related concepts [18, 20]. Although comprehensive, these related concepts, along with Brenner et al.’s (2018)‘s concept analysis [2], did not explicitly include the psychosocial context. Examples of the latter were the “psychosomatic conditions”, as highlighted by family representatives, or stress stemming from the complexity of care or travels. As reported by one stakeholder, travels are a defining element only for those CCHC who access services outside their rural community.

## Recommendations for further concept development and clinical applications

Despite stakeholders’ critiques of McPherson et al. (or MCHB)‘s narrow definition, our findings suggest that varying versions of the commonly used definition of MCHB [11, 31] were reproducible [23]. However, considering contextual and developmental factors [7] helps to better understand CCHC and its related concepts. This is crucial to offer CCHC/families appropriate interactions, ultimately providing them with the needed care or directing them to appropriate services in a timely manner. First, CCHC are still developing, like all youth. Developmental age matters more than their chronological age. In healthy development, it is expected that developmental and chronological ages match. However, there may be a mismatch in some CCHC. Second, there could be a delay or vulnerability in one sphere of development but not another. Third, CCHC’s family context is unique, including the availability of a supportive community.

Our concept analysis has implications for theory, research, and clinical applications, including professional education and practice. Given our comprehensive conceptual and practical analysis, our findings can help to better know CCHC, families, and contextual factors such as siblings, peers, schools, neighborhoods, communities, culture, etc. This paper has practical implications for care providers serving CCHC and their families, including but not limited to psychologists, other mental health clinicians, pediatricians, family physicians, nurses, patient navigators, social workers, professionals of the criminal justice, teachers, and school counselors, among others. A refined understanding of the concept of CCHC is crucial at every clinical stage, from intake, through assessment to delivery of services (e.g., health care, patient navigation, support, education, counseling, and other interventions). Indeed. optimal practices to serve CCHC stemming from a better understanding of this concept may consist of increased efforts to: (1) foster timely and personalized interactions with CCHC and their families, namely at all times and especially around key transition time points (e.g. hospital to home and school, home or school to hospital, pediatric to adult services, and from one developmental stage to another, etc.); (2) ensure an ongoing, efficient communication between families of CCHC and all the professionals involved in their care and/or education; (3) consistently aim for a child/youth- and family-centred approach to care, which focuses more on care needs instead of medical diagnoses, which may or may not be received by CCHC; and (4) to conduct CCHC-related pediatric research to support evidence-based practice, education, prevention, and build theories.

### Strengths and limitations

Our paper used Walker and Avant’s (2011)‘s method [3], which analyzed the literature and information from interviews with stakeholders. The inclusion of the voices of family members and professional stakeholders, from interviews, is a strong contribution to this concept analysis. Indeed, this has likely added another level of rigour to the paper. Furthermore, the addition of the family voice has contributed to the development of the definition by including a refined, patient-and family/caregiver-oriented practical component into this definition. Another strength of the paper operates like a double-edged sword. Specifically, emphasis on the Canadian context is both a strength and a potential limitation, especially for readers operating outside of Canadian communities, even if the literature is drawn internationally.

## Conclusions

### Endorsed definition

The current paper largely confirmed existing definitions whilst building on/expanding the latter by proposing a new iteration as follows: Integrating findings and defining attributes with contextual factors, we define CCHC as involving one or more chronic condition(s), regardless of type(s), whose trajectories are dynamic, requiring services across settings and/or sectors, taking into account severity/intensity of conditions and CCHC’s developmental age whilst being unique to each child and family’s context, often resulting in a lower quality of life. This comprehensive definition can facilitate communication, collaboration, and coordination of care among families, practitioners, researchers, and decision makers. As mentioned earlier, the originality of our paper lies in in the integration of the stakeholders’/family’s input in the development of the definition. As a concept analysis is a work in progress, our findings are provisional. Future research will help to refine the understanding of this concept.

## Data Availability

De-identified data is available upon request of author RA.

## Abbreviations

CCHC: Children with complex health conditions
CMC: Children with medical complexity
CSHCN: Children with special health care needs
CINAHL: Cumulative index to nursing and allied health literature
CCC or CCCs: Complex chronic conditions
CSHCN: Children with special health care needs
ERIC: Education resources information center
FVII deficiency: Factor VII deficiency
HIV/AIDS: Human immunodeficiency virus infection and acquired immune deficiency syndrome
MCBH: Maternal and child health bureau
NB: New Brunswick, an Atlantic Canadian province
PsycINFO: A database of abstracts of literature in the field of psychology
PEI: Prince Edward Island, an Atlantic Canadian province
PubMed: A free search engine accessing primarily the MEDLINE database of references and abstracts of references and abstracts on life sciences and biomedical topics (MEDLINE is the Medical Literature Analysis and Retrieval System Online; a U.S. National Library of Medicine’s life science database)
